# *OsSDS* is essential for DSB formation in rice meiosis

**DOI:** 10.3389/fpls.2015.00021

**Published:** 2015-02-03

**Authors:** Zhigang Wu, Jianhui Ji, Ding Tang, Hongjun Wang, Yi Shen, Wenqing Shi, Yafei Li, Xuelin Tan, Zhukuan Cheng, Qiong Luo

**Affiliations:** ^1^Ministry of Education Key Laboratory of Agriculture Biodiversity for Plant Disease Management, Yunnan Agricultural UniversityKunming, China; ^2^State Key Laboratory of Plant Genomics and Center for Plant Gene Research, Institute of Genetics and Developmental Biology, Chinese Academy of SciencesBeijing, China; ^3^School of Life Sciences, Huaiyin Normal UniversityHuaian, China

**Keywords:** rice, OsSDS, meiosis, DSB formation

## Abstract

SDS is a meiosis specific cyclin-like protein and required for DMC1 mediated double-strand break (DSB) repairing in *Arabidopsis*. Here, we found its rice homolog, OsSDS, is essential for meiotic DSB formation. The *Ossds* mutant is normal in vegetative growth but both male and female gametes are inviable. The *Ossds* meiocytes exhibit severe defects in homologous pairing and synapsis. No γH2AX immunosignals in *Ossds* meiocytes together with the suppression of chromosome fragmentation in *Ossds-1 Osrad51c*, both provide strong evidences that OsSDS is essential for meiotic DSB formation. Immunostaining investigations revealed that meiotic chromosome axes are normally formed but both SC installation and localization of recombination elements are failed in *Ossds.* We suspected that this cyclin protein has been differentiated pretty much between monocots and dicots on its function in meiosis.

## Introduction

Meiosis is one of the key processes in sexual reproduction for all sexually propagating eukaryotic organisms. Meiosis includes one single round of DNA replication but followed by two successive rounds of nuclear segregations (meiosis I and II), and finally produces four haploid gametes with halved chromosomes. The meiotic prophase I is a complicated and prolonged stage, which can be divided into five substages, like leptotene, zygotene, pachytene, diplotene, and diakinesis, based on chromosome characterizations (Ashley and Plug, 1998; Dawe, 1998). During meiotic prophase I, pairing, synapsis and recombination of homologous chromosomes are coordinately accomplished. These events make sure the precise segregation of homologs, and generate both genetic conservation and diverse individuals in the future generations (Zickler and Kleckner, 1999; Page and Hawley, 2003).

In meiosis, DSBs are purposely produced to initiate homologous recombination. The formation of DSBs in meiosis is catalyzed by a type-II topoisomerase-like enzyme Spo11 (Bergerat et al., 1997; Keeney et al., 1997). Meanwhile, a series of cofactors are also required for this process. In budding yeast, the formation of meiotic DSBs requires at least nine other proteins (Rec102, Rec104, Rec114, Mei4, Mer2, Rad50, Mre11, Xrs2, and Ski8) for the cleavage mediated by SPO11 and further broken end resection (Paques and Haber, 1999; Keeney, 2001, 2008). The budding yeast Mre11-Rad50-Xrs2 (MRX) complex is homologous with the mammalian Mre11-Rad50-Nbs1 (MRN), which is required to incise the 5′ end of the break and then form 3′ single-strand tails (Symington, 2002; Mimitou and Symington, 2009). After that, one 3′ free single-strand DNA end recruits two RecA homologs, Rad51 and Dmc1, to mediate the single-end invasion (SEI) with its homologous duplex DNA (Bishop, 1994; Hunter and Kleckner, 2001), and the other 3′ free single-strand DNA end on the other side of the nick is captured simultaneously to form the Double Holliday Junction (DHJ). And then, the DHJ is exclusively processed into crossovers (COs), which represents the accomplishment of homologous recombination (Allers and Lichten, 2001; Bishop and Zickler, 2004; Borner et al., 2004). Consequently, meiotic DSBs are finally repaired during this process.

The function of SPO11 initiating meiotic recombination seems to be widely conserved within eukaryotes, as more and more homologs of SPO11 were identified in a wide range of organisms covering yeasts, flies, mice, humans, and plants (Dernburg et al., 1998; McKim and Hayashi-Hagihara, 1998; Celerin et al., 2000; Hartung and Puchta, 2000; Romanienko and Camerini-Otero, 2000; Grelon et al., 2001; Yu et al., 2010). Unlike animals and fungi where a single SPO11 is sufficient for meiotic DSBs formation, higher plants always possess multiple SPO11 homologs (Keeney et al., 1997; Grelon et al., 2001; Hartung et al., 2007; Shingu et al., 2012; Sprink and Hartung, 2014). But not every SPO11 homolog has the function to cleavage double-strand DNA and generate DSBs in plants. *Arabidopsis* owns three SPO11 homologs and they appear to function in two distinct processes, AtSPO11-1 and AtSPO11-2 in DSB formation, while AtSPO11-3 in DNA replication (Stacey et al., 2006; Shingu et al., 2012). While in monocot rice, there are five SPO11 homologs have been identified (Jain et al., 2006, 2008). Among them, only OsSPO11-4 has been proved to be with double-strand DNA cleavage activity (An et al., 2011). OsSPO11-1 is essential for homologous pairing, recombination and SC installation (Yu et al., 2010; Luo et al., 2014). So, it seems that the formation of meiotic DSBs is more complicated in plants.

Besides SPO11, several other DSB formation proteins have been identified recently in multicellular eukaryotes. Mei1 and Mei4 were shown to be required for DSB formation in mice (Libby et al., 2003; Kumar et al., 2010). Using a high throughput genetic screen, AtPRD1, AtPRD2, and AtPRD3 were identified to be essential for DSB formation in *Arabidopsis* (De Muyt et al., 2009). Nevertheless, AtDFO was also found to be necessary for DSB formation in *Arabidopsis* (Zhang et al., 2012). Studies in rice revealed that CRC1 works together with PAIR1 as a complex to regulate meiotic DSB formation (Miao et al., 2013).

Studies in budding yeast demonstrated that cyclin-dependent kinase Cdc7 and Cdc28 can directly regulate the meiotic DSB formation via the phosphorylation of Mer2 (Henderson et al., 2006; Sasanuma et al., 2008; Wan et al., 2008). However, in plants, only a few cyclins have been found involved in meiosis (Bulankova et al., 2013). The meiosis specific cyclin SDS was first found in *A. thaliana*, which play a specific role in regulating synapsis in prophase I (Azumi et al., 2002; Wang et al., 2004). In a recent study, SDS was found to be required for DMC1-mediated DSB repair (De Muyt et al., 2009). Although the rice *SDS*-RNAi plants showed the similar meiotic defects with those in *Arabidopsis* (Chang et al., 2009), the molecular mechanism of SDS in rice meiosis remains to be clear. Here, we identified the SDS homolog in rice by map-based cloning. Surprisingly, we found OsSDS is essential for DSB formation during rice meiosis, which is much different from that in *Arabidopsis*. We suspected this cyclin protein had been differentiated pretty much between monocots and dicots on its function in meiosis.

## Materials and methods

### Plant materials

The rice (*Oryza sativa* L.) spontaneous mutant *Ossds-1* was isolated from an *indica* rice, Zhongxian 3037. The F2 and F3 mapping populations were generated by crossing the *Ossds-1*^±^ heterozygous plants with a *japonica* cultivar, Zhonghua 11. The other two mutant alleles, *Ossds-2* and *Ossds-3*, both were spontaneous mutants arose in tissue culture of Nipponbare. The meiotic mutant *Osrad51c* has been reported previously (Tang et al., 2014). The *Ossds-1 Osrad51c* double mutant was generated by crossing the two heterozygous *Ossds-1*^±^ and *Osrad51c*^±^, and further identified from the F2 progeny. All plant materials were grown in paddy fields in the summer in Beijing or in the winter in Hainan.

### Molecular cloning of *OsSDS*

Total 861 sterile plants segregated from the F2 and F3 mapping populations were used for isolation the target gene. Sequence-tagged site (STS) markers were developed according to sequence differences between the japonica variety Nipponbare and the indica variety 9311, using the data published on the NCBI website (http://www.ncbi.nlm.nih.gov). All primers are listed in Supplemental Table 1.

### RNAi analysis

In the first exon, a 336-bp fragment of *OsSDS* cDNA sequence was chosen and amplified with the primers SDS-RNAi-F (adding a *BamH*I site) and SDS-RNAi-R (adding a *Sal*I site) (Supplemental Table 1). RNAi vector construction and transformation were performed as described (Wang et al., 2009).

### Complementation test

The complementary plasmid was constructed by cloning the 10.8 kb OSJNBa0081P02 genomic DNA fragment containing the entire *OsSDS* coding region into the pCAMBIA-1300 vector. A control plasmid, containing 7.8 kb of the truncated *OsSDS* gene was also constructed. Both of these plasmids were transformed into EHA105 and then into embryonic calli of *OsSDS*^±^ plants. The genotypes of the transgenic plants were further identify using the Primers SDS-JD (Supplemental Table 1).

### Real-time PCR for transcript expression assay

Total RNA was extracted from the root, internode, leaf and panicle of Zhongxian 3037. Real-time PCR analysis was performed using the Bio-Rad CFX96 real-time PCR instrument (Bio-Rad, http://www.bio-rad.com/) and EvaGreen (Biotium, http://www.biotium.com/). The RT-PCR was carried out using the gene-specific primer pairs SDS-RT-F and SDS-RT-R for *OsSDS*. The primers Ubi-RT-F and Ubi-RT-R for ubiquitin were used as an internal control for the normalization of RNA sample. The results were analyzed using OPTICON MONITOR 3.1 (Bio-Rad). Each experiment had three replicates.

### Cloning the full-length *OsSDS* cDNA

Total RNA was extracted from the panicle of Zhongxian 3037. The 3′ RACE and 5′ RACE were performed according to the protocol of the kit (3′-Full RACE Core Set and 5′ -Full RACE Core Set; Takara, http://www.takara-bio.com/). 3′ RACE was carried out using primers 3R-1F, 3R-2F, 3R-3F, and adaptor primer (P-ada). During 5′RACE, the RNA was reverse transcribed with 5′ (P)-labeled primer (SDS-4Rb); the first and second PCRs were performed using two sets of *OsSDS* specific primers (5R-1 and 5R-2). The 3′ RACE-PCR and 5′ RACE-PCR products were cloned and sequenced. OsSDS amino acid sequence translation and alignment were completed with the Vector NTI 11.5 (Invitrogen). *OsSDS* gene structure diagram was generated from GSDS (http://gsds.cbi.pku.edu.cn/index.php).

### Meiotic chromosome preparation

Young panicles with appropriate size of both *Ossds* mutants and wild type were collected, fixed in Carnoy's solution (ethanol:glacial acetic, 3:1) and stored at −20°C. Microsporocytes at the appropriate meiotic stage were squashed and stained with acetocarmine. After washing the chromosome preparations with 45% acetic acid and freezing them in liquid nitrogen, the coverslips were quickly removed with a razor blade and the slides harbored with samples were dehydrated through an ethanol series (70, 90, and 100%) for 5 min each and finally air-dried. Chromosomes on slides were counterstained with 4, 6-diamidinophenylindole (DAPI) in an anti-fade solution (Vector Laboratories, Burlingame, CA, USA). Finally, images were captured under the ZEISS A2 fluorescence microscope with a micro CCD camera (Zeiss, http://www.zeiss.de/en).

### Fluorescence immunolocalization

Fresh young panicles (40–60 mm) were fixed with 4% (w/v) paraformaldehyde for 30 min at room temperature. Anthers with appropriate stage were squashed into one drop of 1×PBS solution added on a slide. Then, covering the slide with a coverslip and pressing it with appropriate strength, the slide together with the coverslip was frozen thoroughly in liquid nitrogen. After quickly prizing up the coverslip, the slide was dehydrated through an ethanol series (70, 90, and 100%). The following immunolocalization procedure was performed as described (Tang et al., 2014).

The polyclonal antibodies against γ-H2AX, OsMSH5, OsREC8, PAIR2, PAIR3, ZEP1, OsMER3, and OsZIP4 used in this study have been described previously (Wang et al., 2009, 2010; Shao et al., 2011; Shen et al., 2012; Zhang et al., 2012; Luo et al., 2013; Miao et al., 2013).

## Results

### Characterization of a sterile mutant

We identified a spontaneous mutant exhibiting complete sterility in a rice field of Zhongxian 3037. From the heterozygous plant progeny related to this mutation, the normal fertile plants and the sterile plants were segregated in a 3:1 ratio, indicating that it was a single recessive mutation (χ^2^ = 0.57; *P* > 0.05). The mutant plant was normal during vegetative growth and could not be distinguished from the wild type based on plant morphology (Supplemental Figure 1A). However, when come into reproductive stage, its spikelets exhibited complete sterility (Supplemental Figure 1B). So we further examined the mature pollen viability of the mutant by staining with 1% iodine potassium iodide solution (I_2_-KI) (Supplemental Figures 1C,D). Only empty and shrunken pollen grains were observed in the mutant plant, indicating that microspores of the mutant are all abnormal and inviable. Moreover, when pollinated with wild-type pollens, the mutant spikelets still did not set any seeds, indicating that its female gametes were also affected.

### Map-based cloning of *OsSDS*

**Figure 1:**
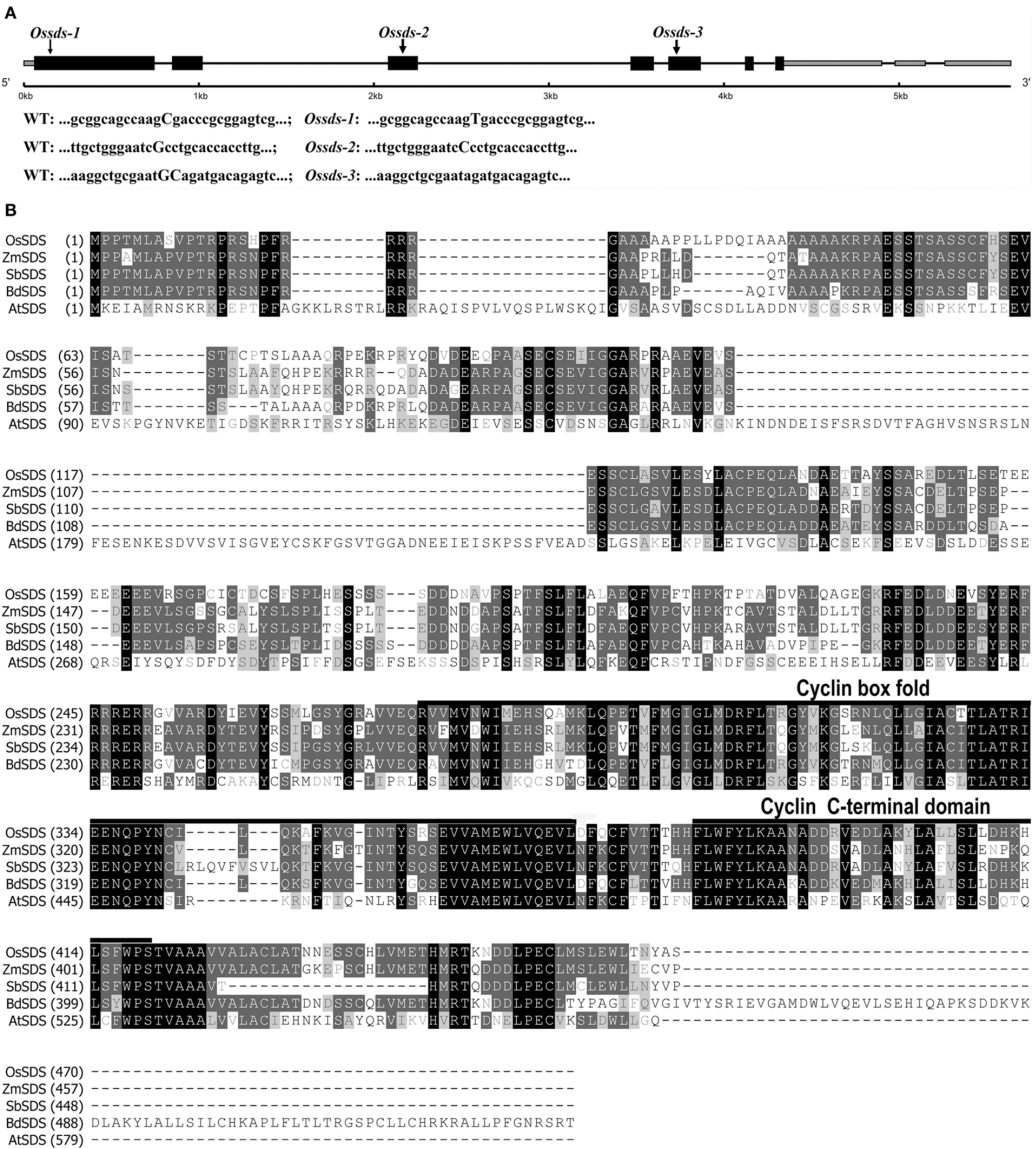
**Gene structure of *OsSDS* and sequence alignment of SDS proteins. (A)** Schematic of representation of gene structure and three mutation sites in *OsSDS*. Exons are represented by black boxes, intones are represented by black lines, 5′UTR and 3′UTR are represented by gray boxes. The capitalized bases in sequences of wild type and mutants represent accurate mutation sites of the three allelic mutants. **(B)** Multiple alignment of SDS protein sequences from different organisms. The numbers at the left of the sequences are the amino acid numbers. The black boxes represent identical sequences; the dark gray boxes represent conservative sequences; the light gray boxes represent weakly similar sequences. A predicted cyclin box fold domain (276–366 amino acids) and a predicted cyclin C-terminal domain (378–419 amino acids) are underlined.

To isolate the mutated gene, we constructed a population by crossing heterozygous plants with a japonica cultivar Zhonghua 11. A total of 861 sterile plants segregated from the F2 and F3 populations were used for mapping the target gene. Linkage analysis initially mapped the target gene onto the long arm of chromosome 3, which subsequently further delimited to a 130 kb region. Within this region, we found one candidate gene (*Os03g12414*) annotated as a putative cyclin with high similarity with *SOLO DANCERS* (*SDS*) gene in *Arabidopsis*. Thus, this candidate gene was chosen to be amplified and sequenced. Sequencing analysis revealed that there was a transversion happened from base C to T in the first exon, which produces a premature termination codon (TGA) and causes early termination (Figure 1A). We named the mutant here *Ossds-1* and suspected the mutation of *Os03g12414* leading to the sterile phenotype. We also obtained two other alleles arose from tissue culture of Nipponbare, named *Ossds-2* and *Ossds-3*, respectively. They all showed the same defects as *Ossds-1*. Sequencing analysis showed that there were a transversion from base G to C at the third exon causing corresponding amino acid A replaced by P in *Ossds-2* and two bases (GC) deletion at the fifth exon resulting in frame-shift mutation causing a premature termination codon (TGA) in *Ossds-3* (Figure 1A). As earlier termination close to the start codon in *Ossds-1* compared with the other mutants, it was selected for the subsequent experiments described blow. To further confirm the mutant phenotype was resulted by the mutation of *OsSDS* gene, RNA interference (RNAi) approach was carried out to down-regulate *SDS* in rice variety Yandao 8. We got 27 transgenic plants with 19 plants exhibited complete sterility. Additionally, the transformation of plasmid pCAMBIA-1300 containing the whole *OsSDS* gene was successful in rescuing the sterility of the mutant plants, just as respected. These results strongly confirmed that the mutation of *OsSDS* gene led to the sterile phenotype as described above.

Expression of *OsSDS* was also analyzed by real-time PCR (RT-PCR). As shown in Supplemental Figure 2, transcripts were all detected at root, internode, leaf and panicle, indicating that *OsSDS* is not a meiosis-specific gene.

### Full-length cDNA cloning and deduced protein sequence of *OsSDS*

The full-length cDNA of *OsSDS* gene was obtained by performing RT-PCR combined with 5′ and 3′ rapid amplification of cDNA ends PCR (RACE-PCR) using specific primers. We found that the *OsSDS* cDNA is comprised of 2786 bp containing an open reading frame (ORF) of 1410 bp and 1376 bp 5′ and 3′ untranslated regions (UTRs). The *OsSDS* cDNA sequence obtained is consistent with one mRNA (GenBank: AK065907.1) from the public network database (http://www.ncbi.nlm.nih.gov/). As shown in Figure 1A, the *OsSDS* gene contains seven exons and six introns. The predicted 469 amino acid protein of OsSDS shares as low as 30.6% identity with SDS in *Arabidopsis*, but with high similarity at the C-terminal (Figure 1B). Compared with the dicots *Arabidopsis*, OsSDS shares more than 60% identity with those in monocots, such as *Zea mays*, *Sorghum bicolor*, and *Brachypodium.* Conserved domain searching in NCBI revealed there are two conserved domains in SDS, namely, cyclin box fold domain (residues 276–366) and cyclin C-terminal domain (residues 378–419) (Figure 1B). Blast searching in NCBI revealed that SDS is a plant specific protein and owns one single copy in the plant kingdom.

### Meiotic defects in *Ossds*

**Figure 2:**
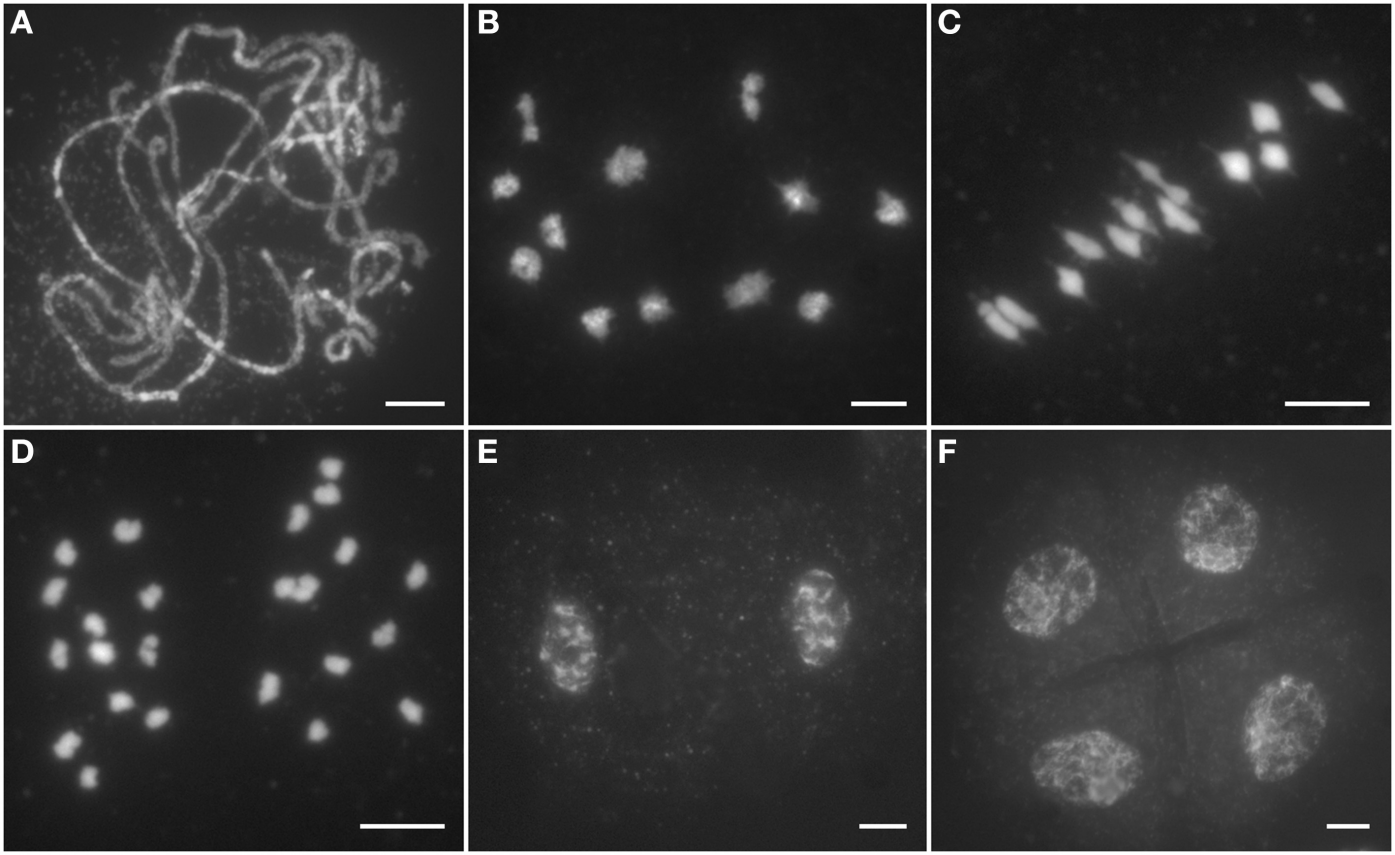
**Male meiosis of the wild type. (A)** Pachytene; **(B)** Diakinesis; **(C)** Metaphase I; **(D)** Anaphase I; **(E)** Dyad; **(F)** Tetrad. Chromosomes stained with 4,6-diamidino-2-phenylindole (DAPI). Bars = 5 μm.

To clarify whether the sterile phenotype of *Ossds* is caused by meiosis defects, chromosome behaviors at different stages of pollen mother cells (PMCs) from both wild type and *Ossds-1* mutants were investigated. In wild type, chromosomes began to condense and became visible as thin thread-like structures at leptotene. After that, homologous chromosomes started to pair at zygotene. Fully synapsis between homologs formed at pachytene (Figure 2A). After further chromosome condensation, 12 bivalents were clearly observed at diakinesis (Figure 2B). Accompanying with the spindle installation, these bivalents aligned on the equatorial plate at metaphase I (Figure 2C). Then, homologous chromosomes separated and moved to the two opposite poles from anaphase I to telophase I (Figures 2D,E). During meiosis II, sister chromatids of each chromosome separated from each other and finally tetrads formed (Figure 2F).

**Figure 3:**
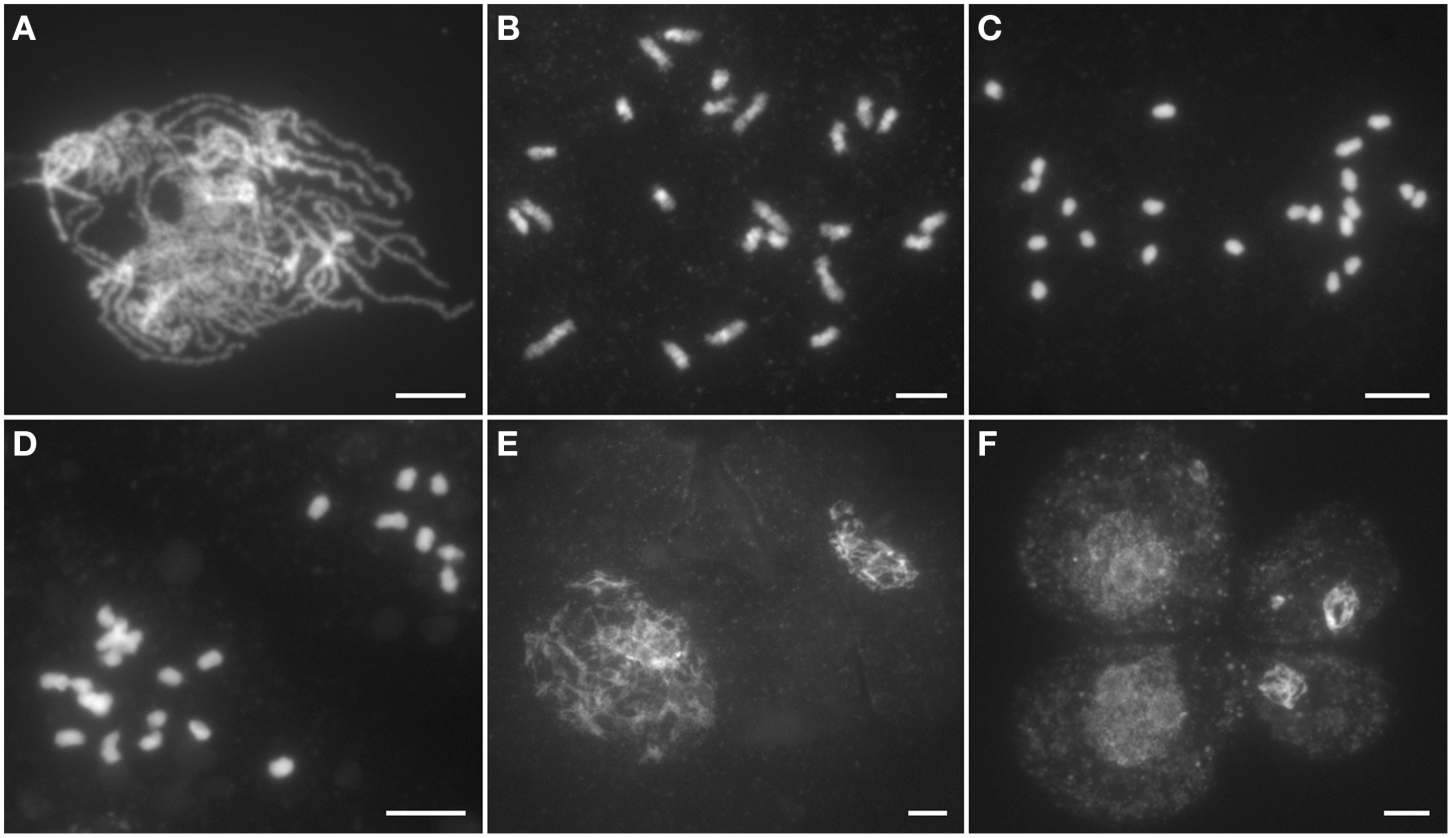
**Male meiosis of the *Ossds-1* mutant. (A)** Pachytene; **(B)** Diakinesis; **(C)** Metaphase I; **(D)** Anaphase I; **(E)** Dyad; **(F)** Tetrad. Chromosomes stained with DAPI. Bars = 5 μm.

In *Ossds-1* PMCs, chromosome behaviors were similar to the wild type from leptotene to zygotene. However, obvious abnormities were first observed at pachytene stage, where the *Ossds-1* mutant shows severely defects in homologous chromosome pairing and synapsis, and fully synapsed homologs were never observed (Figure 3A). Due to the lack of chromosome pairing, only univalents were observed at diakinesis (Figure 3B). During metaphase I, the univalents were unable to align on the equator plate (Figure 3C). From anaphase I to telophase I, they randomly segregated into two daughter cells (Figure 3D). In meiosis II, dyads and tetrads always exhibited different sizes caused by uneven chromosome segregation (Figures 3E,F). Cytological observation of meiocytes from the other two alleles, *Ossds-2* and *Ossds-3*, as well as *OsSDS* RNAi plants showed the same meiotic defects as described in *Ossds-1* (Supplemental Figure 3). Therefore, we proposed that the sterility of *Ossds* was caused by uneven segregation of homologous chromosomes without pairing and recombination during propose I.

### *OsSDS* is essential for meiotic DSB formation

**Figure 4:**
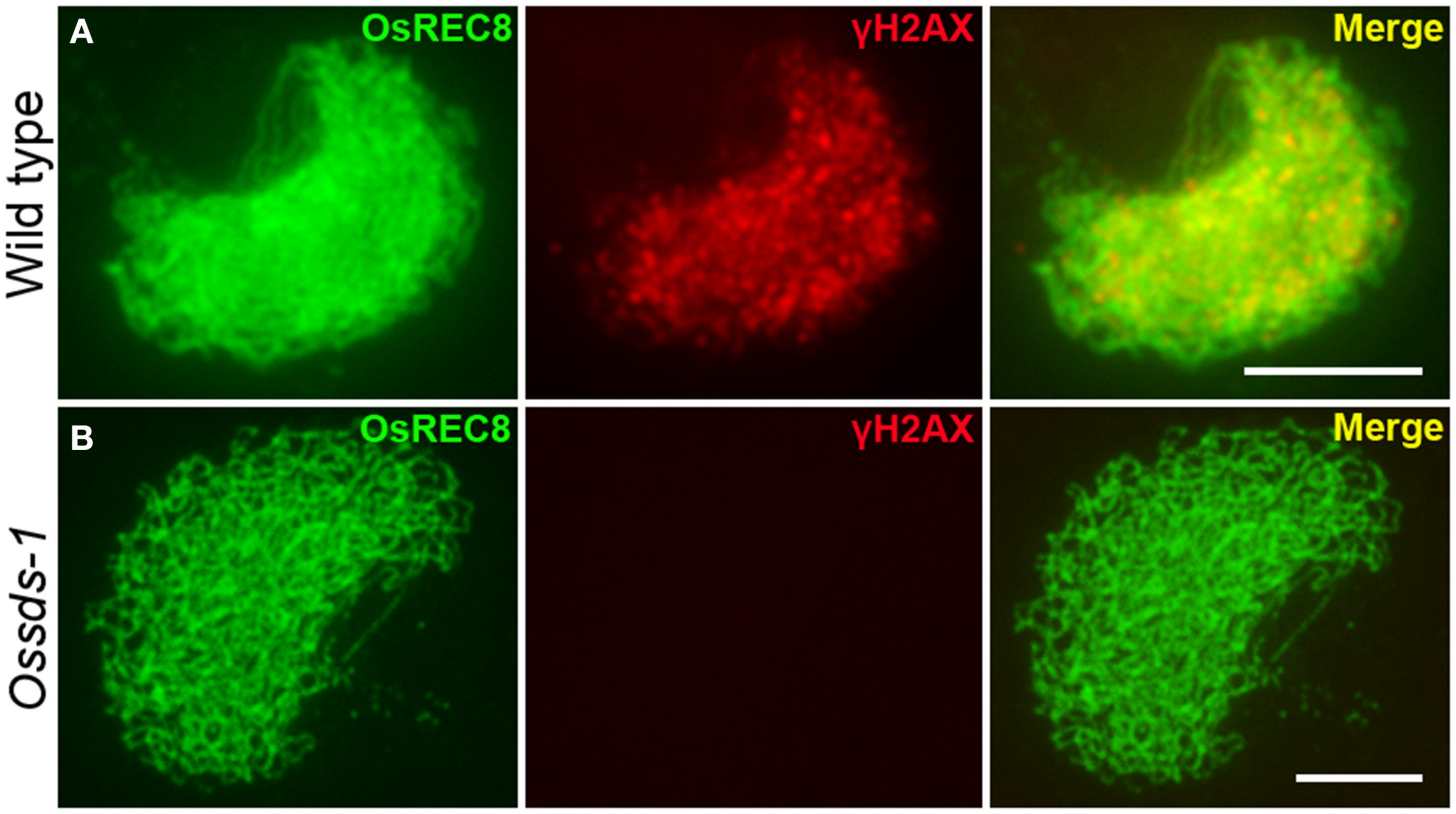
**Immunostaining of γ-H2AX at zygotene in the wild type and *Ossds-1* mutant**. OsREC8 signals were used to indicate the chromosome axes. Bars = 5 μm.

The above cytological observation indicated *Ossds* showed similar defects with the loss of function of CRC1, a meiotic DSB formation protein in rice (Miao et al., 2013). We wondered whether OsSDS is also required for meiotic DSB formation. Phosphorylation of histone H2AX occurs within a few minutes after a DSB initiated in mitosis (Banath et al., 2010), and the same kind phosphorylation takes place during the meiotic DSB formation (Dickey et al., 2009). To verify this hypothesis, a dual-immunostaining experiment was carried out utilizing antibodies specific for γH2AX and OsREC8 raised from rabbit and mouse, respectively, in the meiocytes both from wild type and *Ossds-1*. OsREC8, one of the key cohesion proteins in rice meiosis, is localized onto meiotic chromosomes from leptotene to metaphase I (Wang et al., 2009). Here, we used it as a biomarker to indicate the rice meiotic chromosomes in the prophase I specifically. Results showed that numerous dot and patchy immunosignals of γH2AX were detected at zygotene in wild type (Figure 4A). However, no γH2AX immunosignals was detected in *Ossds-1* meiocytes at the corresponding stage (Figure 4B), showing that OsSDS is essential for meiotic DSB formation.

**Figure 5:**
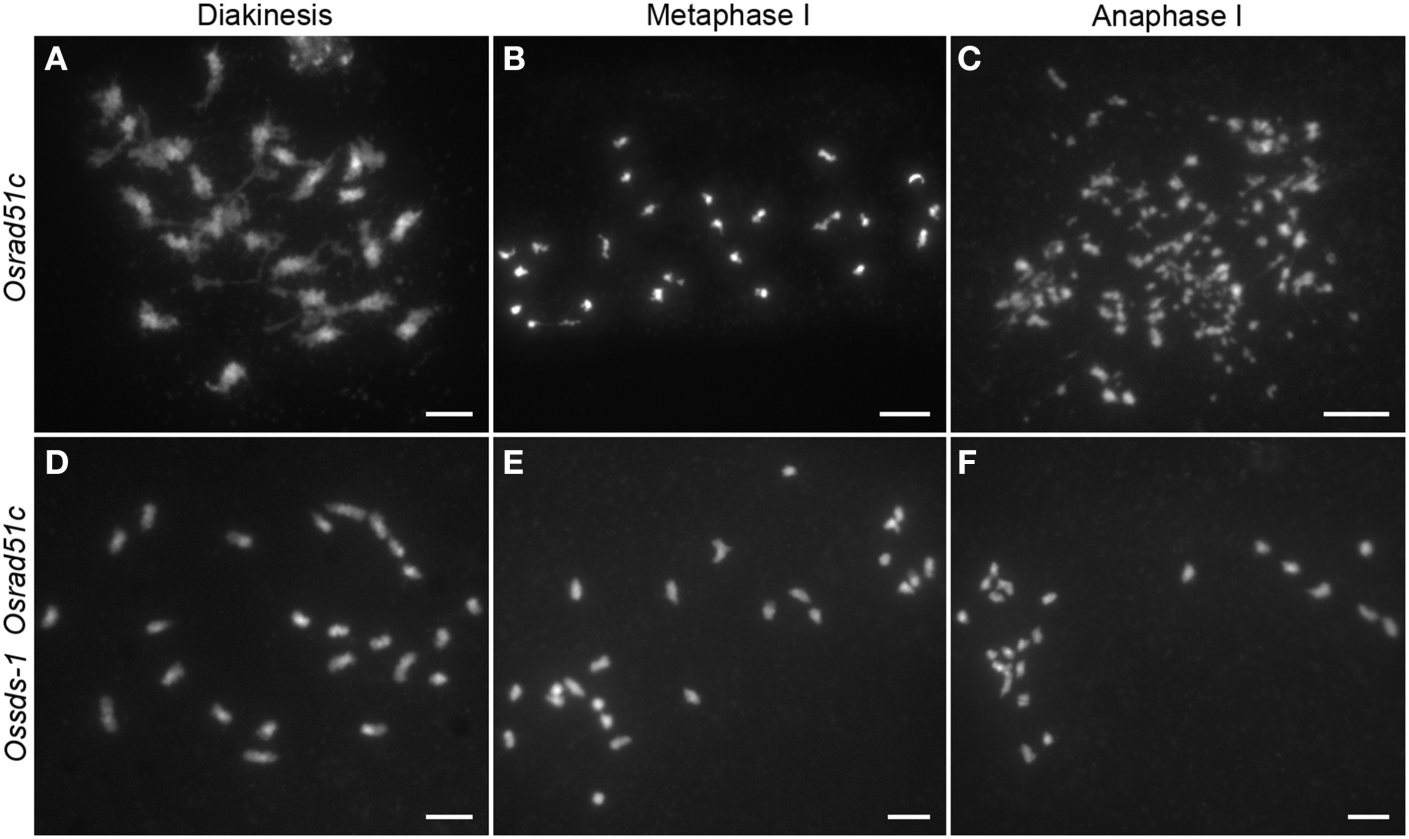
**Comparison of chromosome behaviors between *Osrad51c* and the *Ossds-1 Osrad51c* double mutant**. Chromosomes were stained with DAPI. Bars = 5 μm.

During meiosis, all DSBs will be repaired by the repair system for maintaining genome stability. The loss function of repair proteins always results in chromosome fragmentation. OsRAD51C has been proved to be involved in meiotic DSB repair in rice (Tang et al., 2014). To verify this speculation, the *Ossds-1 Osrad51c* double mutant was generated by crossing the two heterozygous *Ossds-1*^±^ and *Osrad51c*^±^, and further identified from their F2 progeny. In the *Osrad51c* mutant meiocytes, cytological observation shows 24 irregularly univalents appeared at diakinesis (Figure 5A). These univalents did not align well along the equatorial plate, and several chromosome fragments started to be shown at metaphase I (Figure 5B). Numerous chromosome fragments detained at the equatorial plate, while those massive chromosome bodies with centromeres moved into the two opposite poles at anaphase I (Figure 5C). While in the *Ossds-1 Osrad51c* meiocytes, chromosome behaviors were very much similar to *Ossds-1* at the corresponding stages (Figures 5D–F). Together with the above γH2AX immunostaining data, we demonstrated very strong evidence that OsSDS is essential for DSB formation during rice meiosis.

### Meiotic chromosome axes normally formed but SC installation failed in *Ossds*

**Figure 6:**
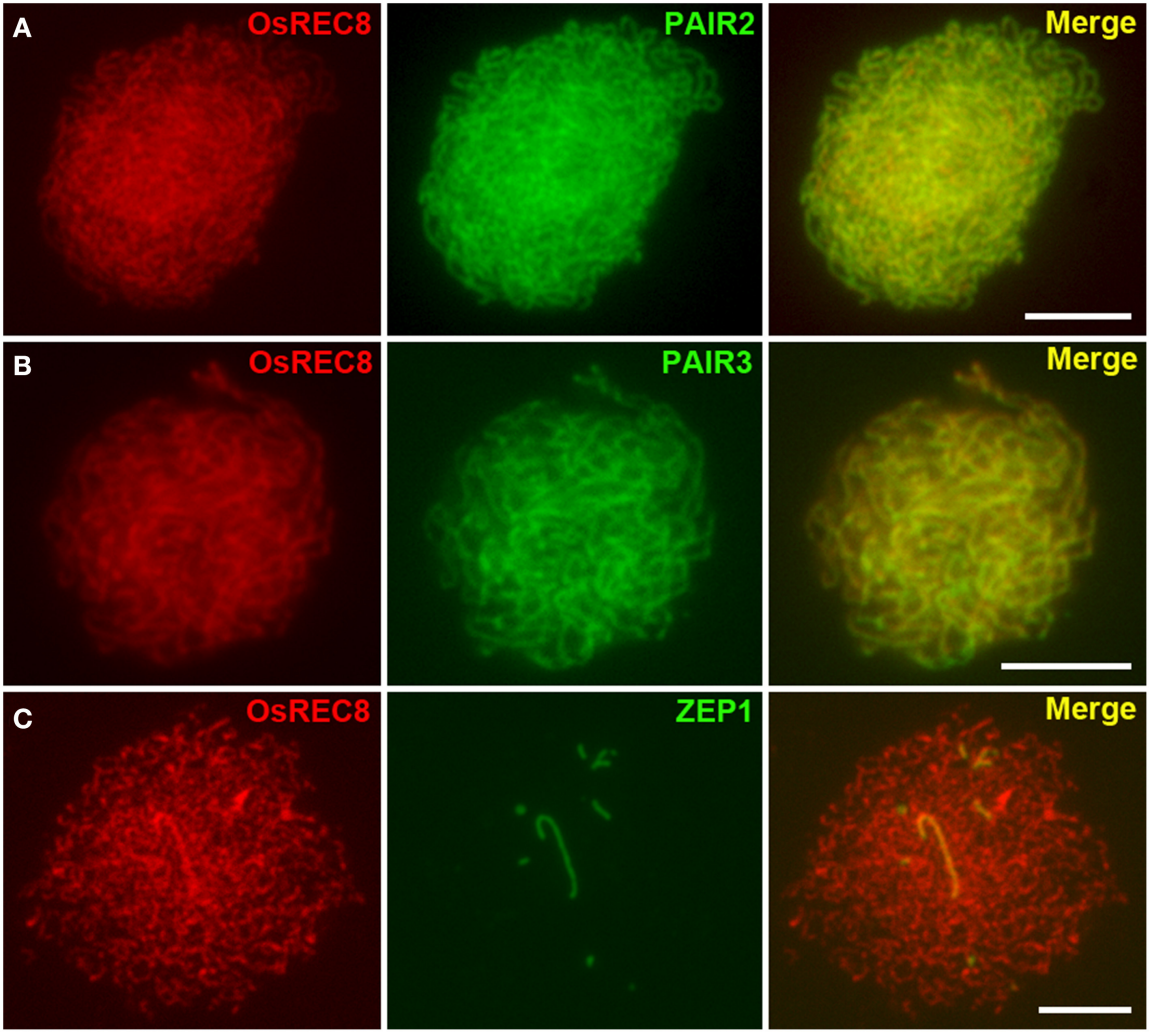
**Dual immunostaining detection of several meiotic proteins in the *Ossds-1*. (A)** OsREC8 (red) and PAIR2 (green) signals at late zygotene; **(B)** OsREC8 (red) and PAIR3 (green) signals at pachytene; **(C)** OsREC8 (red) and ZEP1 (green) signals at pachytene. Bars = 5 μm.

In rice, there are three axis-associated proteins OsREC8, PAIR2 and PAIR3 have been reported playing important roles in SC assembly (Nonomura et al., 2006; Shao et al., 2011; Wang et al., 2011). Except for those axial elements, ZEP1, the central element of SC, has also been identified (Wang et al., 2010). To investigate what kind of SC installation defects happened due to the loss function of OsSDS, we conducted immunodetection experiments using antibodies against PAIR2, PAIR3, and ZEP1 in *Ossds-1* microsporocytes. The results showed that OsREC8 was normally localized onto chromosomes in *Ossds-1* meiocytes. Moreover, both PAIR2 and PAIR3 were overlapped very well with OsREC8 at zygotene, indicating their normal localization along the chromosome axis (Figures 6A,B). However, ZEP1 signals always appeared as short dots or discontinuous lines at early prophase I (Figure 6C), showing the deficient central element installation of SC in the mutant. Thus, the meiotic chromosome axes normally formed but SC installation failed in *Ossds.*

### *OsSDS* is critical for faithful localization of recombination elements onto meiotic chromosomes

**Figure 7:**
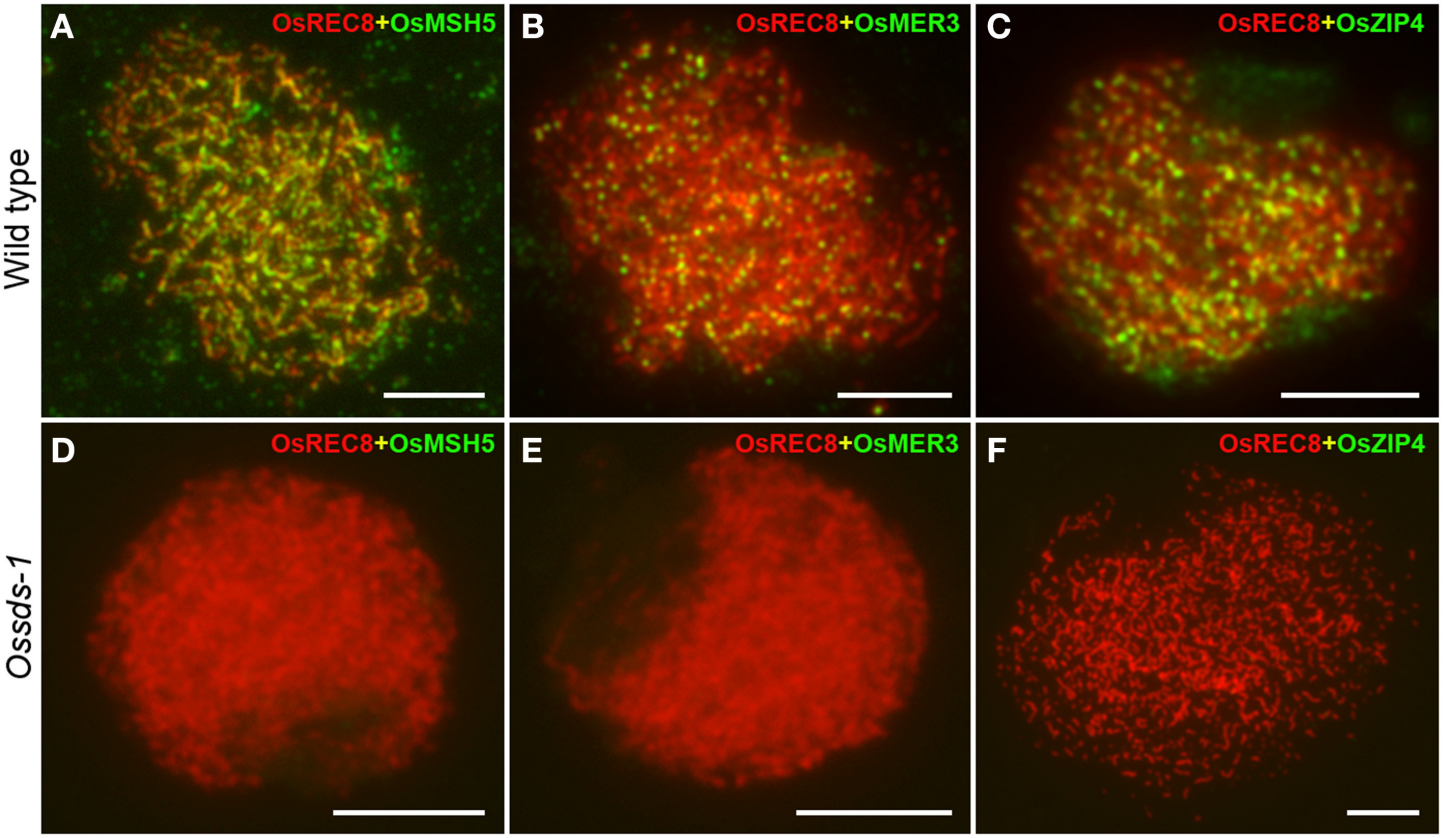
**Immunostaining detection of three ZMM proteins in the wild type and *Ossds-1*. (A–C)** Immunostaining for OsMSH5, OsMER3, and OsZIP4 at zygotene in the wild type. **(D–F)** Immunostaining for OsMSH5, OsMER3, and OsZIP4 at zygotene in *Ossds-1*. OsREC8 was used indicating chromosome axes. Bars = 5 μm.

Meiotic homologous recombination finally falls into two recombination pathways by forming two type crossovers, interference-sensitive COs (class I) and interference-insensitive COs (class II). ZMM proteins are closely associated with class I crossovers formation (Chen et al., 2008; Shinohara et al., 2008). In rice, OsMSH5, OsMER3 and OsZEP4 are three ZMM proteins participating in the class I COs formation (Wang et al., 2009; Shen et al., 2012; Luo et al., 2013). To determine whether the defective OsSDS also affects the localization of OsMSH5, OsMER3, and OsZEP4, dual immunolocalization were carried out using antibodies against OsMSH5, OsMER3, and OsZEP4. In the wild-type microsporocytes, immunostaining experiments showed that OsMSH5, OsMER3, and OsZEP4 were observed as punctuate foci on chromosomes at zygotene (Figures 7A–C), and these foci persisted until late pachytene stage (Wang et al., 2009; Shen et al., 2012; Luo et al., 2013). However, no obvious signal foci of OsMSH5, OsMER3, and OsZEP4 were observed on chromosomes in *Ossds-1* microsporocytes at the corresponding stage (Figures 7D–F), indicating that OsSDS is critical for the localization of OsMSH5, OsMER3 and OsZEP4.

## Discussion

During meiosis, Spo11-catalyzed DSB formation is a highly conserved biological process among eukaryotes (Li and Ma, 2006). As increased data on plant meiosis research, several DSB formation proteins have been identified in higher plants, such as PRD1, PRD2, PRD3, and AtDFO in *Arabidopsis* (De Muyt et al., 2009; Zhang et al., 2012). And in rice, CRC1 was also reported to be involved in meiotic DSB formation besides those OsSPO11 homologs (Miao et al., 2013; Luo et al., 2014). Deficiency of these proteins always cause severe defects in homologous chromosome pairing, synapsis and nondisjunction.

MRE11 is known as an important DSB processing protein. The *Atmre11 sds* double mutant exhibited similar phenotype with the *Atmre11* single mutant (De Muyt et al., 2009), proposing that SDS may be not required for meiotic DSB formation in *Arabidopsis*. Moreover, in *sds* mutant, the localization of DMC1 was abnormal while RAD51 was normally loaded. Thus, the role of SDS in *Arabidopsis* was thought to be necessary for DMC1-mediated DSB repair utilizing the homologous chromosome (De Muyt et al., 2009). While in rice, we provided evidences that OsSDS is essential for meiotic DSB formation. SDS is a plant specific cyclin protein. Amino acid sequences alignment revealed that SDS orthologs between dicots and monocots showed very low identities, *Arabidopsis* SDS sharing 30.6% identity with OsSDS, and as low as 26.1% with BdSDS. While among monocots, they share high identities. For example, ZmSDS shares 69.1% with OsSDS, and 83.1% with SbSDS. The diverged sequences between dicots and monocots suggest that SDS function in meiosis has been differentiated a lot during its evolution.

To date, the precise mechanisms of DSB formation are still unclear. It has been reported that the NBS1 protein is recruited to the end of the DSB soon after a DSB formed, which then initiates the formation of the NBS1/MRE11/RAD50 complex (Kobayashi, 2004). After that, the ATM protein phosphorylates the serine 139 residue of H2AX through its auto-phosphorlation and leading to γH2AX formation (Kinner et al., 2008). Studies revealed that γH2AX plays dual role in the DSB triggered signaling pathway: one is recruiting more MRN complex to the DSB site thus enhancing the signalization by a positive feedback loop; the other one is binding the DNA damage repair proteins (Paull et al., 2000; Minter-Dykhouse et al., 2008). The process of H2AX phosphorlation takes place just within a few minutes after a DSB occurrence (Banath et al., 2010). We did not detect any γH2AX immunosignals in *Ossds*, indicating OsSDS is required for DSB formation during rice meiosis.

It has been reported that cyclin-dependent kinase Cdc7 and Cdc28 can directly regulate the meiotic DSB formation via the phosphorylation of Mer2 in budding yeast (Henderson et al., 2006; Sasanuma et al., 2008; Wan et al., 2008). However, we still do not know the molecular mechanism of how rice cyclin OsSDS being involved in meiotic DSB formation.

### Conflict of interest statement

The authors declare that the research was conducted in the absence of any commercial or financial relationships that could be construed as a potential conflict of interest.
