# Supplementary material for: OsSDS is essential for DSB formation in rice meiosis
Source: Front Plant Sci. 2015 Feb 3;6:21. doi: 10.3389/fpls.2015.00021 (PMC4315026; doi:10.3389/fpls.2015.00021)
Supplement: Supplementary file 1 [file Image1.PDF]

Supplemental Figure 1

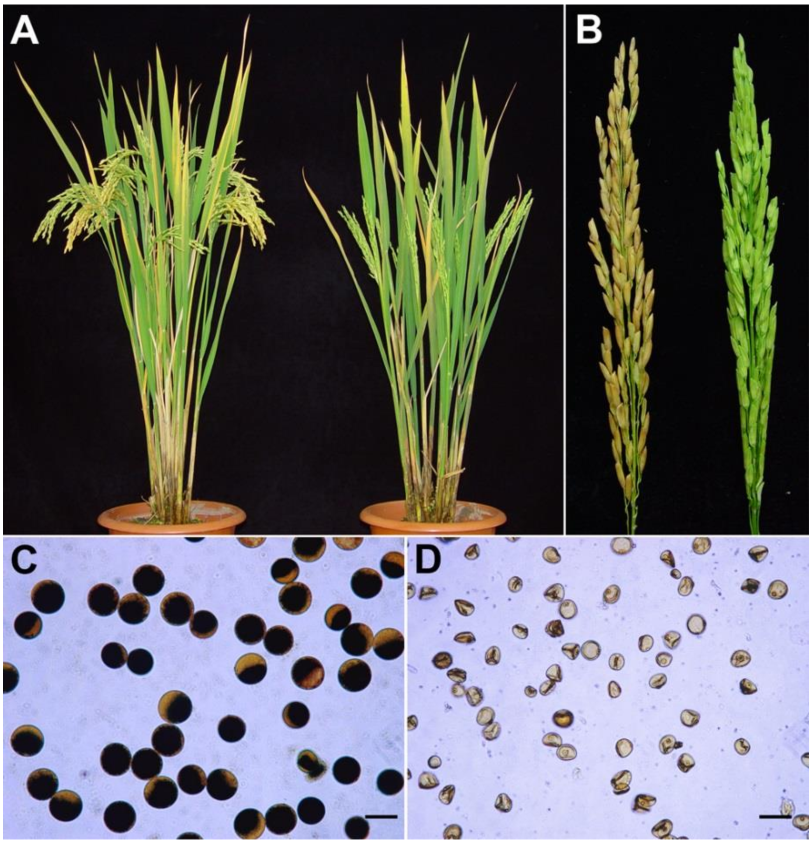

Supplemental Figure 1. Characterization of the *Ossds-1* mutant phenotype. (A) Morphological comparison between a wild type plant (left) and an *Ossds-1* mutant plant (right). (B) Comparison between a wild type panicle (left) and an *Ossds-1* mutant panicle (right). (C) Pollen grains of the wild type plant stained with 1% I_2-KI solution. (D) Pollen grains of *Ossds-1* plant stained with 1% I_2-KI solution. Bars = 50μm.

Supplemental Figure 2

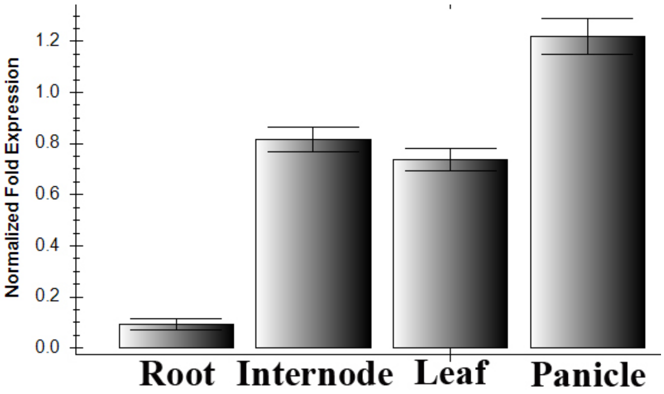

Supplemental Figure 2. Expression analysis of *OsSDS* gene.

Supplemental Figure 3

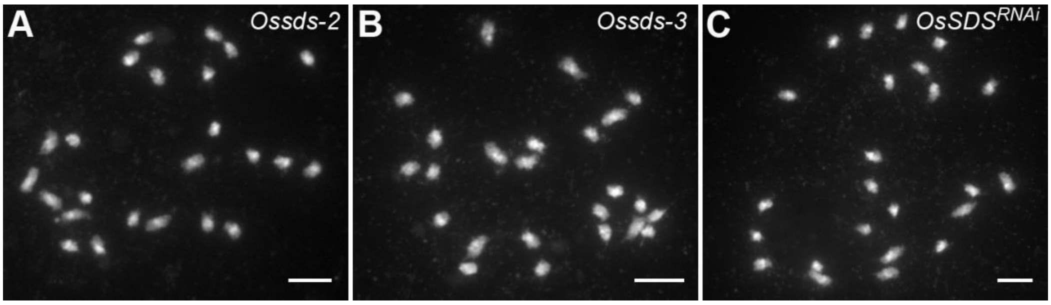

Supplemental Figure 3. Diakinesis chromosomes of *Ossds-2, Ossds-3* and *OsSDS* RNAi plant. Chromosomes stained with DAPI. Bars=5μm.

Supplemental Table 1: Primers designed for *OsSDS* cloning and plasmid construction

Name	Sequence (described 5′to 3′)
MP1-F	CCTGTTCACACCAACAATCC
MP1-R	GTAACACGTACGTATTGATG
MP2-F	GAGATTAGTTCCCAACTGCAA
MP2-R	CGTTAATTATTCAACACAAGT
MP3-F	CGAGTTGCCACCGTAAGGTA
MP3-R	ACTTGGACTGCATACGGCTG
MP4-F	CCTCTCAAATGTTCAAAGCC
MP4-R	TGTAAATGGCATATGGGCTC
MP5-F	AGTGATGACATGGAACGCTG
MP5-R	CAGAATTTCATGATGCGTAG
MP6-F	CCGGGGCTCACCAAATTAAG
MP6-R	GGAATATAACCCTTATGTGGG
MP7-F	GTTCGTGATAGGATATCCATC
MP7-R	GTTCAACTGACAAGTTGCGCC
MP8-F	GTTCTGCAGGTCCCGTATAG
MP8-R	CCATTGTCGTATAGTGCAGAAC
SDS-RT-F	GCTTTCAAAGTAGGGATCAAT
SDS-RT-R	GGTCCTCGACTCTGTCATCTG
SDS-RNAi-F	GAGGATCCGCATCTGCACCGATTGCTCG
SDS-RNAi-R	TCGTCGACAGTTCACCATGACAACGCGC
Ubi-RT-F	CAAGATGATCTGCCGCAAATGC
Ubi-RT-R	TTTAACCAGTCCATGAACCCG
P-ada	CTGATCTAGAGGTACCGGATCC
3R-1F	ACAGCCGAAGTGAGGTCGTC
3R-2F	GCCTTGCTCTCACTTCTGGA
3R-3F	CGCAGTGGTAGCCCTTGCTT
SDS-4Rb	pTCCTCCAATTGGCTCAATAG
5R-1F	CTTCCACAGCGAGGTCATCT
5R-1R	GATCAGGGAGCAGAGGAGGA
5R-2F	ATCTCCGCCACCTCCACCAC
5R-2R	GTCGGCACCGATGCAAGCAT
SDS-JD-F	TCACACACAAAGCACACAGC
SDS-JD-R	ACCTCATTGTCCAAGTCCTC
